# The role of polysomnography in Parkinson's disease: objective sleep assessment and clinical indications

**DOI:** 10.1055/s-0046-1829160

**Published:** 2026-10-23

**Authors:** Alessandra Zanatta, Carlos Mauricio Oliveira de Almeida, Marcus Vinicius Della Coletta, Hélio Afonso Ghizoni Teive

**Affiliations:** 1 Universidade Federal do Paraná, Departamento de Medicina Interna, Curitiba PRBrazil.; 2 Universidade Estadual do Amazonas, Escola de Ciências da Saúde Manaus AMBrazil.

**Keywords:** Sleep Apnea, Obstructive, Parkinson disease, Polysomnography, REM Sleep Behavior Disorder

## Abstract

Sleep disorders (SDs) are highly prevalent in Parkinson's disease (PD), affecting up to 80% of patients during the disease and significantly contributing to impaired quality of life, cognitive decline, motor worsening, fall risk, and increased caregiver burden. Polysomnography (PSG) remains the gold standard for objective sleep assessment in PD and plays a central role in differential diagnosis and therapeutic planning. This review examines the spectrum of SDs identifiable by PSG, including rapid eye movement (REM) sleep behavior disorder (RBD), obstructive sleep apnea (OSA), periodic limb movements in sleep, and alterations in sleep architecture. Video-polysomnography (v-PSG) is essential for confirming REM sleep without atonia (RWA), which is the electrophysiological hallmark of RBD and a recognized prodromal marker of synucleinopathies. Also, PSG quantifies the apnea–hypopnea index (AHI), oxygen desaturation burden, and sleep fragmentation, parameters associated with cognitive impairment and disease progression. While home sleep apnea testing (HSAT, type 3) may be useful for selected cases with high suspicion of OSA, comprehensive in-laboratory (type 1) PSG provides superior diagnostic accuracy, particularly in patients with complex or overlapping sleep disturbances. Integrating objective sleep evaluation into routine PD management is crucial to identify treatable comorbidities, improve prognostic stratification, and optimize motor and non-motor outcomes.

## INTRODUCTION

Parkinson's disease (PD) is a common, chronic, and progressive neurodegenerative disorder characterized by a constellation of motor and non-motor symptoms 1 2 Although motor features define clinical diagnosis, sleep disorders (SDs) are highly prevalent, substantially impair quality of life, and may precede motor manifestations by years or even decades, indicating SDs as early markers of neurodegeneration. 3 4

Overall, up to 80% of individuals with PD experience some form of SDs. Obstructive sleep apnea (OSA), insomnia, rapid-eye movement (REM) sleep behavior disorder (RBD), and Restless Legs Syndrome (RLS) are among the most frequently reported conditions and are associated with worsened clinical outcomes. 5 6

Polysomnography (PSG) provides an objective and quantitative assessment of sleep architecture, respiratory parameters, motor phenomena during sleep, and REM sleep without atonia (RWA), thereby enabling accurate diagnostic characterization. 7 8 9 10 In many cases, clinical history and questionnaire-based assessments are insufficient to differentiate overlapping sleep phenotypes, such as RBD, sleep-disordered breathing (particularly OSA), and periodic limb movements (PLMS) during sleep which frequently coexist within the same patient ( Figure 1 ). 7 8 10

**Figure 1:**
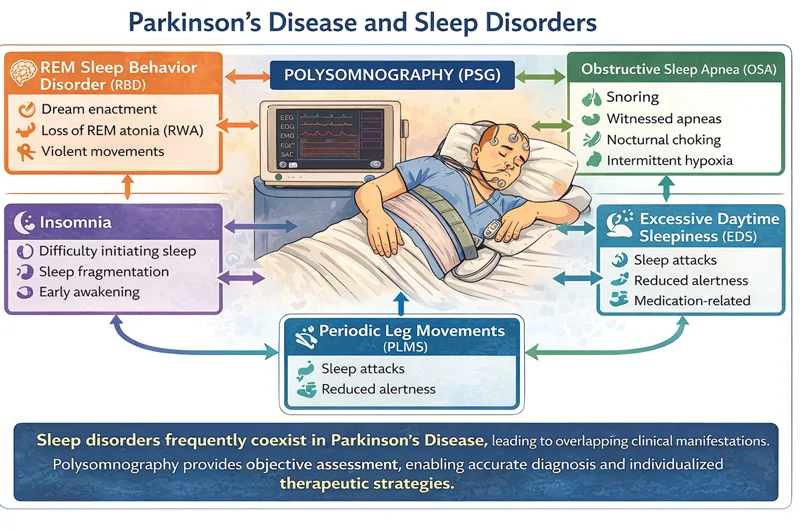
Sleep disorders in PD: clinical manifestations and the diagnostic role of PSG.

Given the high prevalence of SDs in PD and their substantial clinical impact—including increased caregiver burden—accurate recognition and appropriate management are essential competencies for clinicians. Therefore, a comprehensive review of the role of PSG in the evaluation and management of SDs in PD is warranted, with relevance for both neurologists and general practitioners.

## SLEEP DISORDERS IN PARKINSON'S DISEASE

The prevalence of SDs in patients with PD is substantially higher than in the general population. 5 11 Clinical studies indicate that up to 80% of individuals with PD experience some form of SDs during the progression of the disease. Reported prevalence rates include insomnia in 44 to 84% of patients, excessive daytime sleepiness in around 35 to 40%, OSA in 45 to 66%, and RBD in 35 to 46% of cases. 5 6 11 12

This high burden of SDs reflects the multifactorial pathophysiology of PD, including degeneration of neural centers regulating the sleep–wake cycle, a high prevalence of psychiatric comorbidities, the impact of motor and non-motor symptoms on nocturnal sleep, and the adverse effects of dopaminergic and other medications used in disease management. 11

These conditions frequently coexist, generating a complex and overlapping clinical phenotype that limits the reliability of subjective reports and questionnaire-based assessments. This diagnostic complexity underscores the need for objective evaluation, with PSG playing a pivotal role in accurate differential diagnosis and individualized therapeutic planning.

Regarding RBD, it is a parasomnia characterized by the loss of the normal skeletal muscle atonia during REM sleep, resulting in the emergence of complex motor behaviors and dream-enactment episodes during this sleep stage ( Figures 2 3 ). 8 This condition is widely recognized as a well-established prodromal manifestation of Parkinson's disease and other α-synucleinopathies. 3 4 13 Longitudinal studies have demonstrated that the prevalence of sleep disturbances, including probable RBD symptoms, increases as the disease progresses. Importantly, this disorder is one of the strongest clinical predictors of future neurodegeneration.

**Figure 2:**
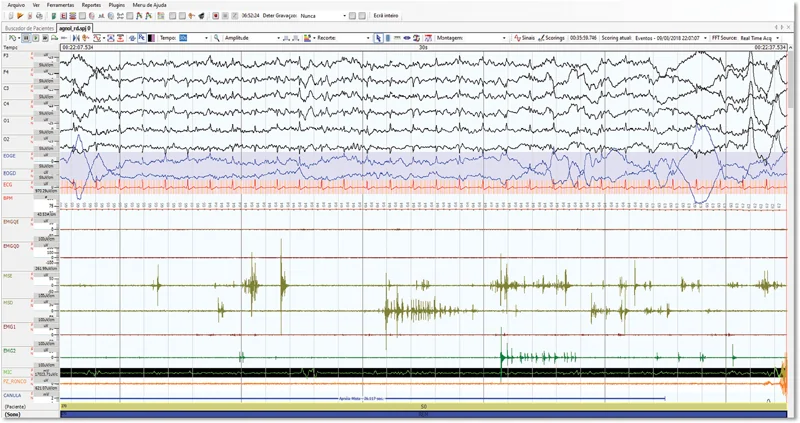
Abbreviations: BPM, heart rate; ECG, electrocardiogram; EOG-E, left electrooculogram; EOG-D, right electrooculogram; EMGE E/D, chin electromyography; MSE, left upper extremity electromyography; MSD, right upper extremity electromyography; EMG 1, left lower extremity electromyography; EMG 2, right lower extremity electromyography; MIC, microphone. PSG epoch illustrating phasic RWA in a patient with PD and RBD.

**Figure 3:**
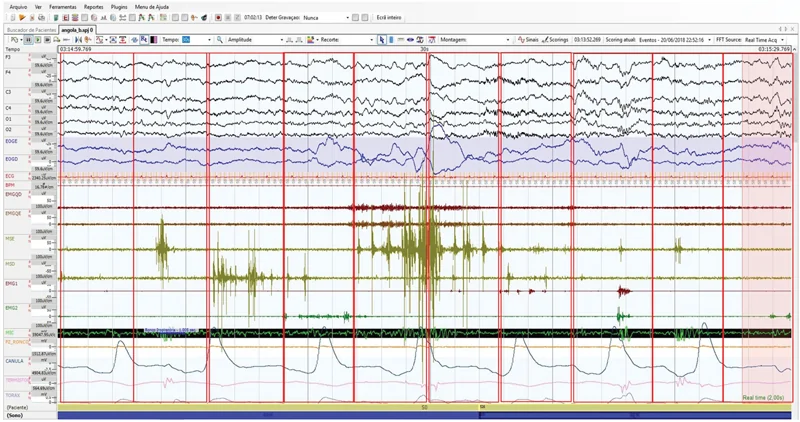
Abbreviations: BPM, beats per minute, heart rate; ECG, electrocardiogram; EOG-E, left electrooculogram; EOG-D, right electrooculogram; EMGE E/D, chin electromyography; MSE, left upper extremity electromyography; MSD, right upper extremity electromyography; EMG 1, left lower extremity electromyography; EMG 2, right lower extremity electromyography; MIC, microphone. Quantification of RWA using the 3-second mini-epoch scoring method during REM sleep in a PD patient.

A meta-analysis reported that more than 90% of individuals with RBD eventually developed a neurodegenerative disorder, with PD accounting for the largest proportion of phenoconversions (44%). 14 Because clinical history alone may be insufficient for diagnosis, dream-enactment behaviors and abnormal motor activity during sleep should be confirmed by video-polysomnography (v-PSG), which demonstrates REM sleep without atonia and remains the diagnostic gold standard. 8

The isolated finding of RWA has predictive value for the future development of RBD and synucleinopathies, but this value is moderate and depends on clinical context and longitudinal follow-up. A cohort study with a mean follow-up of 8.6 years showed that only 7.3% of individuals with isolated RWA progressed to clinical RBD, while 71.4% presented at least “one biomarker of neurodegeneration, such as substantia nigra hyperechogenicity or mild cognitive impairment”. 15 Additionally, electromyographic (EMG) activity increased significantly over time in these individuals, suggesting subclinical progression. Isolated RWA may be one of the earliest markers of neurodegeneration, preceding motor and behavioral symptoms, and its severity may be associated with a higher risk of synucleinopathies. 15

Protocols for diagnosing RWA use quantitative criteria based on PSG with electromyographic recording of the submentalis (SM), flexor digitorum superficialis (FDS), and, in some cases, tibialis anterior muscles. The Sleep Innsbruck Barcelona (SINBAR) criteria recommend analyzing tonic, phasic, and “any” (any muscle activity) EMG activity in 3-second mini-epochs during REM sleep, with specific thresholds: SM “any” ≥ 6.5% and combined SM + FDS “any” ≥ 15.1% are the main cutoffs for high diagnostic accuracy, with area under the curve (AUC) values above 0.95 in populations with idiopathic RBD. 9

The American Academy of Sleep Medicine incorporated the SINBAR criteria into its guideline, recommending the use of these thresholds and the inclusion of multiple muscles to increase sensitivity and specificity. 7 The REM atonia index, an automated metric, also shows performance comparable to visual scoring, although with lower specificity in some populations. Automated methods, such as the RBDtector tool for Python (Python Software Foundation), demonstrate high concordance with manual scoring, particularly for “any” activity in the SM and FDS muscles, facilitating standardization and reproducibility across centers. 16 17

Actigraphy represents a promising outpatient screening tool for RBD by detecting abnormal movement patterns during sleep. 18 These patients exhibit greater overall activity, with movements of shorter duration, higher magnitude, and greater temporal dispersion. Machine learning algorithms applied to actigraphic data achieved AUC values of 0.84 to 0.87 in multicenter validations, and combining actigraphy with symptom questionnaires, such as anosmia, reached 100% specificity and precision with 88% sensitivity. 19 The main advantages of actigraphy over PSG are its low cost and the possibility of home recording over multiple nights, which increases the likelihood of identifying asymptomatic patients. It could be used for large-scale identification of candidates for confirmatory PSG investigation.

Restless legs syndrome (RLS), also known as Willis-Ekbom disease (WED), is a sensorimotor neurological disorder characterized by an irresistible urge to move the lower limbs, often accompanied by unpleasant sensations that worsen during rest, predominate in the evening or at night, and are relieved by movement. 8 20 Its pathophysiology is multifactorial, involving dysfunction of central dopaminergic pathways, disturbances in brain iron homeostasis, glutamatergic dysregulation, and neuronal hyperexcitability. Brain iron deficiency, particularly in the substantia nigra, may impair dopamine synthesis and function, representing a key pathophysiological mechanism. 21

The RLS is more common in patients with PD than in the general population, with a prevalence of approximately 20%, according to a recent meta-analysis including 6,990 individuals from 46 studies. 22 The presence of RLS in PD has been associated with poorer quality of life, greater non-motor symptom burden, and a more severe neuropsychiatric profile. 23 As such, PD may represent an important risk factor for RLS, as both conditions share pathophysiological mechanisms, including dopaminergic dysfunction, alterations in iron metabolism, and involvement of the A11 diencephalospinal pathway. 24

Although dopaminergic therapy can provide symptomatic relief, long-term treatment has been associated with increasing RLS prevalence, rising from 10.6% in patients treated for less than 2 years to 23.1% after 5 years of therapy. Furthermore, augmentation—a progressive worsening of symptoms characterized by an earlier daily onset—may occur in up to 60% of patients receiving levodopa. 24 25

Although the diagnosis of RLS is primarily clinical, PSG may provide valuable complementary information in selected patients, 8 20 allowing the objective detection and quantification of PLMS, the principal motor manifestation associated with RLS, and assesses their effects on sleep architecture, continuity, and efficiency. 7 Although not required for routine diagnosis, PSG may be useful in atypical or refractory cases, aiding in the exclusion of differential diagnoses, identifying coexisting SDs, and providing objective measures of disease severity and treatment response. 24

Circadian dysfunction is a recognized non-motor manifestation of PD, often presenting as an advanced sleep–wake phase characterized by earlier sleep onset and awakening times. 26 27 Its pathophysiology is thought to involve neurodegeneration of structures responsible for circadian regulation, including intrinsically photosensitive retinal ganglion cells, the hypothalamic suprachiasmatic nucleus, and the pineal gland, with α-synuclein deposition likely contributing to these alterations. 26 Beyond sleep–wake disturbances, circadian dysfunction may manifest as loss of the normal nocturnal blood pressure decline, reduced amplitude of core body temperature rhythms, and decreased melatonin secretion. Diagnosis is primarily clinical and may be supported by sleep diaries, chronotype questionnaires, actigraphy, and, in selected cases, assessment of melatonin secretion or its metabolites. 8

Obstructive sleep apnea (OSA) is a sleep-related breathing disorder characterized by recurrent episodes of partial or complete collapse of the upper airway during sleep, resulting in a reduction (hypopnea) or cessation (apnea) of airflow despite persistent respiratory effort. These events lead to intermittent hypoxemia, sleep fragmentation, and activation of multiple neurohumoral pathways, contributing to a broad range of cardiovascular, metabolic, and neurocognitive consequences. 7 8

The gold standard for OSA diagnosis is PSG, which allows objective assessment of respiratory events, sleep architecture, oxygen saturation, and associated physiological parameters. Obstructive apnea is defined as a ≥ 90% reduction in airflow lasting at least 10 seconds despite persistent respiratory effort, whereas hypopnea consists of a ≥ 30% airflow reduction associated with oxygen desaturation or arousal. The diagnosis and severity of OSA are determined by the apnea–hypopnea index (AHI), with it being defined as an AHI ≥ 5 events/h in symptomatic individuals or ≥ 15 events/h regardless of symptoms. Severity is classified as mild (5–14.9 events/h), moderate (15–29.9 events/h), and severe (≥ 30 events/h). 7 8

According to previous studies, OSA affects approximately 20 to 70% of PD patients, compared with 2 to 14% in the general population. 6 28 Population-based cohort studies have suggested that OSA is associated with an increased risk of developing PD. 29 30 31 Notably, a recent nationwide cohort study further demonstrated that OSA independently increases the risk of incident PD, while early continuous positive airway pressure (CPAP) treatment substantially attenuates this risk, highlighting OSA as a modifiable midlife factor in the neurodegenerative cascade. 31 These findings support a potential link between sleep-disordered breathing and neurodegeneration, possibly mediated by intermittent hypoxia, oxidative stress, and neuroinflammatory mechanisms. 32

## SPECIFIC SCALES FOR ASSESSING SLEEP DISTURBANCES IN PARKINSON'S DISEASE

Although PSG remains the gold standard for the objective evaluation of SDs, validated clinical questionnaires play a fundamental role in the initial screening, characterization of subjective symptoms, and assessment of their impact on quality of life in patients with PD. These instruments provide complementary information that cannot be fully captured by PSG, particularly regarding perceived sleep quality, excessive daytime sleepiness, and sleep-related behavioral symptoms.

Several scales have been specifically validated or widely applied in PD populations. Together, these scales represent practical, low-cost, and validated tools for systematic sleep assessment in PD, supporting clinical screening, longitudinal monitoring, and therapeutic decision-making, particularly when used in combination with objective measures such as PSG ( Table 1 ). 33 34 35 36 37 38 39 Three scales stand out for their psychometric properties, clinical applicability, and endorsement by the Movement Disorder Society (MDS): the Parkinson's disease sleep scale-2 (PDSS-2), the scales for outcomes in Parkinson's disease-sleep (SCOPA-Sleep), and the REM sleep behavior disorder screening questionnaire (RBDSQ). 36 37 38 39

**Table 1:** Specific scales for assessing sleep disorders in PD

Scales	Assessment	Score range	Cut-off	Main domains	Clinical utility
**ESS**	Subjective excessive daytime sleepiness	0–24	≥ 10 EDS	Sleep propensity in 8 daily situations	Screening and monitoring daytime sleepiness
**PDSS-2**	Nocturnal sleep disturbances in PD	0–60	Higher scores indicate worse sleep disturbance; ≥ 15 suggests significant impairment	Sleep initiation, maintenance, and nocturnal motor symptoms	PD-specific nocturnal symptom severity assessment
**SCOPA-Sleep**	Nocturnal and daytime sleep problems in PD	Night: 0–15; Day: 0–6	Higher scores indicate worse sleep dysfunction	Night sleep quality and daytime sleepiness	Validated PD-specific sleep outcome measure
**RBDSQ**	Probable RBD	0–13	≥ 5 suggests probable RBD	Dream-enactment behaviors and motor activity during sleep	Screening for RBD
**PSQI**	General sleep quality	0–21	> 5 indicates poor sleep quality	Sleep latency, duration, efficiency, disturbances	Global sleep quality assessment

Abbreviations: ESS, Epworth Sleepiness Scale; EDS, excessive daytime sleepiness; PD, Parkinson's disease; PDSS-2, Parkinson's Disease Sleep Scale version 2; SCOPA-Sleep, Parkinson's Disease-Specific Scale; RBD, REM sleep behavior disorder; RBDSQ, REM Sleep Behavior Disorder Screening Questionnaire; PSQI, Pittsburgh Sleep Quality Index.

The original PDSS was developed to assess nocturnal disturbances in PD. 38 40 Its revised version, the PDSS-2, is a 15-item self-report instrument (score range: 0–60) organized into three domains: disturbed sleep, nocturnal motor symptoms, and Parkinson's disease-related nocturnal symptoms. It demonstrates good psychometric performance, with satisfactory internal consistency (Cronbach's α = 0.73) and test–retest reliability (intraclass correlation [ICC] = 0.80). Validated cut-off scores of ≥18 and ≥15 have been proposed to identify clinically significant sleep disturbances, showing acceptable sensitivity and specificity across different populations. 41 42 The MDS recommends the PDSS-2 for both screening and severity assessment, emphasizing its value in characterizing potential contributors to poor sleep, including RLS, nocturnal akinesia, pain, and sleep-disordered breathing. 43

The SCOPA-Sleep comprises two independent subscales: Nocturnal Sleep (NS) and Daytime Sleepiness (DS) domains, both demonstrating a unidimensional structure, with factor analyses explaining 60 and 57% of the variance, respectively, and good internal consistency (α = 0.82–0.84). 5 A cut-off score ≥ 7 on the NS subscale has been proposed to identify clinically relevant nocturnal sleep disturbances. 39 Unlike the PDSS-2, which explores specific nocturnal symptoms and their potential causes, the SCOPA-Sleep provides a broader assessment of both nighttime sleep problems and daytime somnolence, making it particularly useful when a balanced evaluation of the sleep–wake cycle is required. 39 Although the two instruments show a moderate correlation (r = -0.60), their only partial overlap suggests that they capture complementary aspects of sleep dysfunction in PD, supporting their combined use in comprehensive assessments.

Several questionnaires have been developed to screen RBD, offering adequate sensitivity and specificity for clinical and epidemiological use. However, none can replace PSG, which remains the gold standard for the diagnosis of RBD. To date, the RBDSQ is the only screening questionnaire validated in Brazilian Portuguese. 36 37 This 13-item instrument has shown good psychometric performance, including adequate internal consistency (α = 0.81), high test–retest reliability (ICC = 0.86), and acceptable diagnostic accuracy (AUC = 0.73). In the Brazilian validation study, a cut-off score of ≥4 provided the best balance between sensitivity (84%) and specificity (58%). 37

Collectively, instrument selection should be guided by the clinical or research objective: the PDSS-2 is preferred when an etiological profile of sleep disturbance is required; the SCOPA-Sleep when balanced nocturnal and diurnal assessment is the priority; and the RBDSQ when screening for prodromal synucleinopathy markers. It should also be noted that sleep scale scores in PD may be partially confounded by mood and personality dimensions, and multidimensional modeling approaches may better reflect the latent structure of these instruments than traditional factor analysis alone. 44

## THE DIAGNOSTIC UTILITY OF POLYSOMNOGRAPHY

While patient history and sleep questionnaires are valuable screening tools, they are inherently subjective and often insufficient for a definitive diagnosis, particularly when multiple SDs coexist. Objective measurement is crucial for characterizing the nature and severity of sleep disturbances in PD, and PSG is the definitive gold standard diagnostic tool for this. 7 8 11 A comprehensive PSG study can simultaneously record multiple physiological parameters, providing a detailed and integrated picture of sleep architecture and pathology.

First, respiratory parameters are recorded through continuous monitoring of nasal/oral airflow, thoracic and abdominal respiratory effort, and blood oxygen saturation. This allows for the precise detection of apneas (cessations of breathing) and hypopneas (reductions in breathing). These events are used to calculate the AHI and oxygen desaturation index, which are essential for diagnosing and quantifying the severity of sleep-disordered breathing.

Regarding neurophysiological parameters, the combination of electroencephalography (EEG), electrooculography, and EMG of the chin muscles allows clinicians to objectively divide sleep into its distinct phases of non-REM (N1, N2, N3) and REM sleep. This is critical for assessing sleep architecture and for detecting specific abnormalities. The hallmark PSG finding of RBD is RWA, a pathological increase in muscle tone during REM sleep detected via chin and limb EMG.

Finally, EMG recordings from the legs are used to detect and quantify periodic limb movements during sleep. These data are used to calculate the periodic limb movement index, the number of periodic limb movements per hour of sleep, which is the key objective marker for PLMS.

## A COMPARISON OF POLYSOMNOGRAPHY METHODS: IN-LABORATORY VERSUS HOME SLEEP TEST

Attended in-laboratory PSG (PSG, type 1) remains the gold standard for the comprehensive evaluation of SDs. Alternative approaches include unattended full PSG (type 2), usually performed at home, and simplified cardiorespiratory monitoring techniques (types 3 and 4). Importantly, Types 3 and 4 are not true PSG studies because they lack EEG recording and, therefore, cannot assess sleep architecture. Consequently, they are more appropriately classified as cardiorespiratory monitoring devices.

Understanding the strengths and limitations of each modality is particularly important in PD, where multiple SDs frequently coexist. In such cases, full PSG (type 1 or 2) is often required, as simplified monitoring cannot detect key neurophysiological abnormalities, including RWA and alterations in sleep architecture ( Table 2 ).

**Table 2:** A comparison of PSG types (types 1–2) and cardiorespiratory monitoring test (types 3–4)

Feature	Type 1	Type 2	Type 3 (HSAT)	Type 4 (HSAT)
**Number of channels**	≥ 7 channels (EEG, EOG, EMG, ECG, airflow, respiratory effort, SpO _2_ , video)	≥ 7 channels (same as type 1 PSG)	4–7 channels (airflow, respiratory effort, SpO _2_ , heart rate ± snoring/position)	1–2 channels (usually SpO _2_ ± airflow or heart rate)
**Location**	Sleep laboratory (attended, in-clinic)	Home (unattended)	Home (unattended)	Home (unattended)
**Sleep staging**	Yes (EEG-based sleep staging)	Yes	No	No
**Clinical indications**	Full evaluation: OSA, RBD, PLMS, parasomnias	Same as type 1 PSG, but unattended and normally without video	Primarily OSA diagnosis	OSA screening only
**Accuracy**	Gold standard for diagnosis of all sleep disorders	High diagnostic accuracy, but increased risk of signal loss due to unattended recording	Moderate accuracy for OSA diagnosis, but it may underestimate severity	Lower accuracy; s uitable only for screening purposes
**Ability to diagnose RBD**	Yes (requires EMG and video monitoring)	No. Normally performed without video	No	No
**Cost & accessibility**	Highest cost; limited availability	Moderate cost; improved accessibility	Lower cost; widely available	Lowest cost; primarily a screening tool

Abbreviations: ECG, electrocardiography; EEG, electroencephalography; EMG, electromyography; EOG, electrooculography; HSAT, home sleep apnea testing; OSA, obstructive sleep apnea; PLMS, periodic limb movements in sleep; RBD, REM sleep behavior disorder; SpO _2_ , peripheral oxygen saturation.

According to the American Academy of Sleep Medicine, home sleep apnea testing (HSAT) may be used as an alternative to in-laboratory PSG for diagnosing OSA in uncomplicated adults with a high pretest probability of moderate-to-severe disease. However, PSG remains the preferred diagnostic modality in patients with significant cardiorespiratory disease, neuromuscular disorders, hypoventilation syndromes, chronic opioid use, prior stroke, severe insomnia, or suspected comorbid SDs. Additionally, a negative, inconclusive, or technically inadequate HSAT in the setting of persistent clinical suspicion of OSA should be followed by in-laboratory PSG. 7

### Type 1–Polysomnography (in-laboratory study)

#### *Definition and key features*

Type 1 is the comprehensive gold standard test and is typically performed in a sleep laboratory. This method involves simultaneous monitoring of a wide range of physiological channels, including EEG, electrooculography, chin and limb EMG, electrocardiography (ECG), respiratory airflow and effort, and blood oxygen saturation (oximetry).

Standardized criteria like the Manual for the Scoring of Sleep and Associated Events are employed to score this exam. Often, vPSG is used, combining PSG with video to document behavioral events. 7 8

#### *Advantages in PD*

Confirmation of RBD requires type 1 PSG to definitively verify the core diagnostic feature: RWA. The report should describe the percentage of REM epochs that meet the criteria for RWA. 7 9 Comprehensive sleep architecture provides detailed measures of sleep macrostructure (e.g., REM percentage). Furthermore, differential diagnosis helps rule out conditions that mimic RBD, such as OSA, non-REM-sleep parasomnias, nocturnal epileptic seizures, and PLMS. 10

Also, accurate quantification allows precise measurement of respiratory parameters, such as the AHI and respiratory disturbance indexes; and REM-sleep parameters, such as the REM atonia index. 7

#### *Disadvantages in PD*

One of the main disadvantages of type 1 in PD is the laboratory effect, when the unfamiliar environment causes poor sleep quality, though this is a general limitation. Furthermore, the lack of mobility/comfort caused by PD motor symptoms, such as rigidity and tremor, can make the laboratory setting and wire placement uncomfortable for this population.

### Type 2–Unattended full polysomnography (home-based study)

#### *Definition and key features*

Type 2 PSG provides the full complement of physiological channels typically recorded in type-1 studies; however, it is conducted in the patient's home environment and without overnight technical supervision. This modality preserves the ability to perform comprehensive sleep staging and to assess respiratory events and motor phenomena in a more naturalistic setting, potentially mitigating first-night effects and other laboratory-related influences on sleep architecture.

#### *Advantages in PD*

The main advantage of type 2 in PD is ecological validity, which due to home-based recording, may better reflect habitual sleep patterns in patients with PD, minimizing environmental disruption.

Furthermore, its comprehensive assessment maintains the ability to evaluate sleep macrostructure, respiratory parameters (e.g., respiratory disturbance index), and motor events, such as periodic limb movements and RWA. The greater accessibility may facilitate the exam for patients with mobility limitations or advanced motor symptoms who face challenges traveling to a sleep laboratory. Finally, reduced laboratory burden avoids potential discomfort associated with prolonged laboratory stays.

#### *Disadvantages in PD*

On the other hand, the lack of real-time supervision due to the absence of a technologist increases the risk of signal loss, electrode displacement, and technical artifacts, particularly in patients with tremor or dyskinesias. Potential data incompleteness caused by technical failures may necessitate repeat studies, impacting cost-effectiveness and patient adherence.

Also, behavioral documentation is limited, since video recording is often unavailable, reducing diagnostic certainty for RBD, especially when confirmation of RWA and associated behaviors is required.

### Type 3–Home sleep apnea test (HSAT)

#### *Definition and key features*

Type 3 devices are limited-channel recordings primarily designed for diagnosing OSA in the home setting. They typically monitor respiratory parameters, heart rate, and oximetry, but generally do not include EEG for sleep staging or EMG for RWA detection. 7 8

#### *Advantages in PD*

Its feasibility/comfort allows testing in the patient's natural environment, which may be beneficial given the mobility issues often experienced by PD patients. As such, if OSA screening is the primary clinical suspicion, a type 3 device may suffice. 45

#### *Disadvantages in PD*

Type 3 PSG is inadequate for PD-specific disorders and insufficient for diagnosing RBD because it lacks the necessary EMG channels to measure RWA. Incomplete sleep assessments cannot accurately measure objective sleep architecture. Since PD involves complex non-motor symptoms and central sleep control pathology, a comprehensive study (type 1) is often preferred.

### Type 4–Home sleep apnea test (HSAT)

#### *Definition and key features*

Type 4 studies are simplified, unattended home recordings that typically monitor one or two physiological signals—most commonly pulse oximetry, with or without airflow. They do not include EEG; therefore, they cannot provide sleep staging or accurate total sleep time measurement. Respiratory indices are estimated based on recording time, which may underestimate event frequency. 45

#### *Advantages in PD*

Type 4 devices are simple and accessible, particularly for patients with mobility limitations. These have minimal wiring, which improves comfort and tolerability, and has lower cost and technical complexity. They're a useful screening tool for suspected moderate-to-severe OSA.

#### *Disadvantages in PD*

- On the other hand, this device does not assess sleep architecture and cannot diagnose RBD, RWA, PLMS, not be used for the AHI.

## Future directions

Advances in computational analysis and machine learning are expanding the potential applications of sleep recordings. Large datasets derived from PSG signals can now be analyzed using artificial intelligence approaches capable of identifying disease-specific patterns.

A recent study introduced a multimodal sleep foundation model that can predict multiple diseases using large-scale sleep recordings. 46 These approaches suggest that sleep signals may eventually serve as predictive biomarkers for neurological and systemic diseases. For PD, such methods may improve early detection and risk stratification.

In conclusion, sleep disorders are highly prevalent and clinically relevant in PD, contributing to worse motor and non-motor outcomes and reduced quality of life. The PSG remains the gold standard for objective evaluation, enabling accurate diagnosis of conditions such as OSA, RBD, and PLMS, which frequently coexist and may not be reliably identified through clinical assessment alone.

Rather than supporting indiscriminate routine use, PSG for PD should be guided by clinical context and integrated into a systematic sleep assessment strategy, particularly when there is diagnostic uncertainty, suspected overlapping SDs, treatment failures, or the need for objective confirmation.
